# The changing epidemiology of leptospirosis in Israel.

**DOI:** 10.3201/eid0706.010611

**Published:** 2001

**Authors:** R Kariv, R Klempfner, A Barnea, Y Sidi, E Schwartz

**Affiliations:** Chaim Sheba Medical Center, Tel Hashomer, Israel.

## Abstract

We reviewed all serologically confirmed cases of leptospirosis from 1985 to 1999 in Israel, where the disease is endemic. There were 59 cases, with an average annual incidence of 0.05/100,000. The dominant serogroup, Leptospira icterohemorrhagica, occurred in 29% of patients; in an earlier study (1970-1979), it accounted for only 2%. Serogroups that occurred mainly in rural areas accounted previously for 79% but had declined to 32%.


### The Changing Epidemiology of Leptospirosis in Israel

**Revital Kariv,*† Robert Klempfner,*† Ada Barnea,‡ Yechezkel Sidi,*† and Eli Schwartz*†**
*Chaim Sheba Medical Center, Tel Hashomer, Israel; †Sackler School of Medicine, Tel Aviv University, Tel Aviv, Israel; and ‡Israel Institute for Biological Research, Ness Ziona, Israel

We reviewed all serologically confirmed cases of leptospirosis from 1985 to 1999 in Israel, where the disease is endemic. There were 59 cases, with an average annual incidence of 0.05/100,000. The dominant serogroup, *Leptospira icterohemorrhagica*, occurred in 29% of patients; in an earlier study (1970-1979), it accounted for only 2%. Serogroups that occurred mainly in rural areas accounted previously for 79% but had declined to 32%.

Leptospirosis, a zoonotic disease caused by a spirochete, is often related to occupation. Humans are infected through contact with animal reservoirs or a contaminated environment (soil, sewage, or water). Several animal species (rats, mice, or hedgehogs) are natural reservoirs of the disease, while humans are a dead-end host. *Leptospira icterohemorrhagica* is transmitted by rats and is found in sewage water. *L. canicola*, which is usually transmitted by dogs but rarely by cattle and swine, is common among field and irrigation workers. *L. hardjo*, which is transmitted mainly by cattle, is commonly found in dairy workers (1). The clinical spectrum of the disease depends on the serogroup and the host, ranging from a mild flulike illness to severe disease with multiple organ failure (Weil's disease).

Since 1950, Israel has been considered endemic for leptospirosis (1,2) with a peak incidence of 3.6/100,000 in the 1960s (3). From 1970 to 1979, 251 cases of leptospirosis were diagnosed, with a reported attack rate of 0.2/100,000, mainly in agricultural areas (1). Since 1979, no epidemiologic study has reported the prevalence of different serogroups and the epidemiologic pattern of the disease. We have reviewed all known cases of leptospirosis in Israel from 1985 through 1999.

### The Study

In Israel, leptospirosis is a notifiable disease, and reported cases are investigated by the Department of Epidemiology of the Ministry of Health (MOH). For each case, a brief medical report was submitted to the MOH, and an epidemiologic investigation was carried out by an epidemiologic nurse. We reviewed charts at the MOH and serologic information at the central laboratory and extracted data including serologic results, demographic information, residential area, occupation, risk factors, and outcome.

Cases were considered related to occupation if patients were farmers, veterinary doctors, sewage workers, or cattle or swine workers, all occupations known to be associated with leptospirosis. Cases were considered "inner-city related" if the patient had no obvious occupation or activity known to be a risk factor for infection. Residential area was defined as urban or rural.

### Diagnosis

All serologic investigations were done by the microscopic agglutination test. Twenty-two reference serovars of living spirochetes were used, with 20 pathogenic (*L. interrogans)* and two nonpathogenic (*L. biflexa)* serovars.[1] A laboratory-confirmed case was defined as a fourfold increase in antibody titer or a single titer $\geq$1:200.

During the study period, 1985-1999, 59 cases of leptospirosis were serologically confirmed (60% based on the first single serum and the rest on paired sera). Ages of these patients ranged from 16 to 66 years (mean 42 ± 15 years); 53 (90%) were male. Cases occurred throughout the year with no clear seasonality.

The dominant serovars were *L. icterohemhorragica* with 17 cases, followed by *L. hardjo* (12 cases) and *L. balum* (12 cases). The disease was related to occupation in 28 cases, mostly in farmers, including pig farmers and dairy workers. In 19 cases exposure was in the inner city, usually in markets (Table). Most of these cases (13 of 19) were due to *L. icterohemorrhagica*, and the patients were either shopkeepers or occasional shoppers in the markets. The rest of *L. icterohemorrhagica* cases were also from an urban setting, mainly Tel Aviv, but these affected sewage workers, a known risk for leptospirosis. *L. habdomadis* group (serovars *hardjo* and *swajisak*) and *L. gripotyphosa* affected mainly farmers. Information about exposures was not available for eight patients. In our series, one case of *L. icterohemorrhagica*

Address for correspondence: Eli Schwartz, Department of Medicine "C" and Center for Geographic Medicine, Chaim Sheba Medical Center, Tel Hashomer 52621, Israel; fax: + 972 5302011; e-mail: elischwa @post.tau.ac.il

[1]*Leptospira* serovars tested: Serovars of *L. interrogans*: Ictero copenhagi Weinberg, Javanica Vcldrat–ATCC 233479, Canicola Hond Utrecht IV-ATCC 2347, Australis–ATCC 23605, Grippothyphosa Moskow V-ATCC 23469, Cynopteri Canazone, Sejroe M-84, Pyrogenes–ATCC 23480, Szwajizak Szwajizak, Ballum Castelloni–ATCC 23580, Mini Sari, Burgas, Hardjo, Ballum Mus, Pomona-ATCC 23478, Tarassovi–ATCC 23481, Bataviae ATCC, Sejreo Bratislava, Rachmat-ATCC 23603, Ictero RGA –ATCC 43642
Serovars of *L. biflexa*: Patoc, Andamana

Table. Characteristics of patients with leptospirosis

| Serovar | No. of cases | Sex | | Living area | | Source of Infection | | Occupation[a] |
|---|---|---|---|---|---|---|---|---|
| | | M | F | Urban | Rural | Inner-city | Occupation | |
| *Leptospira icterohemorrhagica* | 17 | 16 | 1 | 17 | 0 | 13 | 4 | S: 3<br>B: 1 |
| *L. hardjo* | 12 | 11 | 1 | 1 | 10 | 0 | 8 | C: 7<br>B: 1 |
| *L. swajisak* | 4 | 3 | 1 | 2 | 2 | 1 | 1 | S: 1 |
| *L. ballum* | 12 | 10 | 2 | 5 | 7 | 4 | 8 | F: 6<br>S: 2 |
| *L. canicola* | 7 | 7 | 0 | 3 | 4 | 1 | 5 | Sw:4<br>S: 1 |
| *L. gripophytosa* | 3 | 3 | 0 | 0 | 3 | 0 | 2 | F: 1<br>Sw:1 |
| *L. cinopteri* | 1 | 1 | 0 | 1 | 0 | N.A. | N.A. | |
| Mixed | 3 | 2 | 1 | N.A. | N.A. | N.A. | N.A.. | |

[a] Occupations at risk: S = sewage contact; Sw = swine-related occupation; F = ordinary farmers; C = cattle and dairy farmers; B =butcher; NA = information not available.

infection was fatal, for a case-fatality rate of 5.8% among patients infected with *L. icterohemorrhagica*. The clinical manifestations were severe hepatorenal involvement and death after massive cerebral hemorrhage.

Comparison of this period with the earlier report from 1970-1979 (1) shows that serogroups such as *L. habdomadis* and *L. grypotyphosa*, which are associated with farming and had been the dominant pathogens, accounting for 55% and 25% of cases, respectively, had declined to 27% and 5%. The urban serovars of *L. icterohemorrhagica* became the dominant groups, increasing from 2% to 29% (Figure 1).

### Conclusions

Over the last 15 years, several epidemiologic characteristics of leptospirosis in Israel have changed: attack rate, affected population, and dominant pathogenic serogroups. The reported attack rate in Israel has declined from 2 to 3.6/100,000 during 1950-1970, to 0.2/100,000 during the 1980s, and approximately 0.05/100,000 during our study period (Figure 2). This trend is most likely due to improved sanitation and increased awareness of risk factors for the disease. Although underreporting and underdiagnosis cannot be ruled out, the ratio between total serologic tests requested for leptospirosis and the rate of positive results during the last 15 years is extremely low: 1.4% compared with 8% during the 5 years before our study. These data may indicate a higher awareness of leptospirosis among physicians in Israel and do not suggest underdiagnosis.

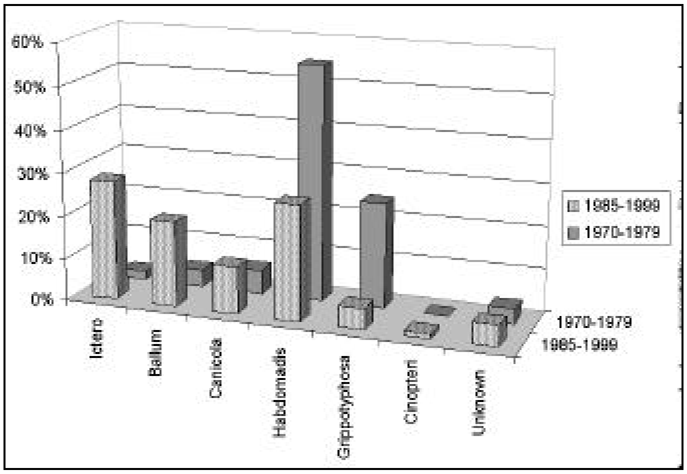

Figure 1. Comparison of *Leptospira* serogroups in Israel

The disease was once more common in rural and agricultural areas and was related to farming. The last report, from 1970 to 1979, showed almost all cases to be rural, while during our study period most cases were urban (mostly in Tel Aviv).

The environmental changes in Israel were associated with a marked change in the epidemiology of pathogenic serogroups. The incidence of the *L. habdomadis* serogroup was 25 cases per year (1), but declined to <1 case per year in our study period (Table). The vectors associated with these groups are cattle and rodents, and therefore farmers and agriculture workers were affected. A recent study in a farming area in Israel where cattle were found to be infected with *L. habdomadis* showed that all 50 farmers in the area who were working with infected animals were seronegative for leptospirosis (A. Barnea, unpub. data). These data may support the assumption that awareness of the disease among these high-risk populations has increased, leading to the use of gloves while in contact with animals. The change to mechanization of field cultivation also prevents contact with animal excreta and thus may reduce leptospira infection.

Over the last few decades, Israel's population has grown rapidly (due to massive immigration), and a trend toward rapid urbanization may also have shifted the disease to the cities. In urban areas, *L. icterohemorrhagica* is the dominant pathogen that causes multisystem involvement (Weil's disease) with a high reported case-fatality rate. This was the main infecting serogroup during our study period, accounting for 29% of all cases, compared with 2% during the previous study. Nonetheless, the absolute number of infected subjects did not change substantially over the last 50 years: During the period 1948 to 1968, there were a dozen cases (2); from 1968 to 1982 there were 14 cases (2); and our study (1984-1999) identified an additional 17 cases. All

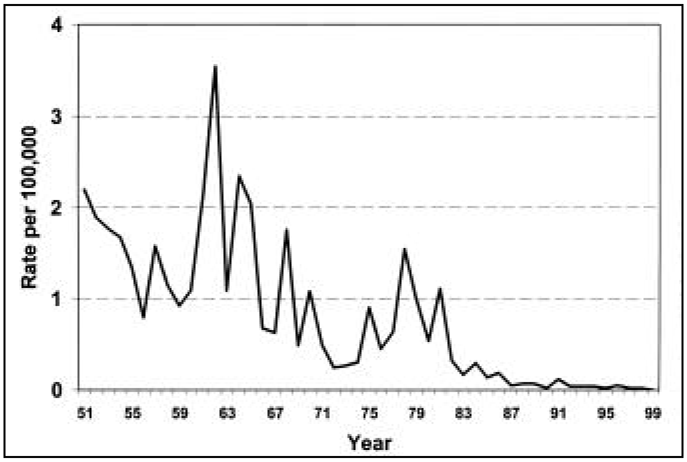

Figure 2. Incidence (rate per 100,000) of leptospirosis in Israel from 1951 to 1999 (adapted from ref. 3, with permission).

*L.icterohemorrhagica* cases were in an urban area, mainly in Tel Aviv, affecting workers in the city's largest market. The vector associated with transmission of *L. icterohemorrhagica* is *Rattus norvegicus*. The last survey of rats in these areas, in 1982, revealed an infection rate of 37% (4), indicating a need for better sanitation control.

There are almost no large-scale reports on the epidemiologic characteristics of the disease in industrialized countries: a report from Ireland during 1990-1996 revealed an annual incidence of 1.2 cases/100,000 (5). In the United States, the annual rate from 1988 to 1994 was approximately 0.02/100,000 (6). Two recent reports from Europe have shown a shift in the epidemiology of the disease, from being an occupational disease towards a disease associated with recreational activities, including travel to tropical countries (7,8). Our case series included only one patient in whom we suspected that the disease was imported (Thailand).

The main change in pattern of the disease in Israel was the decline of occupational-agricultural-related disease and persistence of foci in large cities. Inner-city foci causing sporadic urban leptospirosis have also been described in the United States (9), with *L. icterohemorrhagica* the dominant pathogen. A recent report from Brazil described a large urban epidemic, mainly of *L. icterohemorrhagica* (90% of cases), with a case-fatality rate of 15%, despite aggressive intervention (10). In Brazil leptospirosis had been a sporadic rural disease, but with urbanization and population growth a new environment for urban transmission has been created, mainly in slums and areas lacking proper sanitation (10). Israel exemplifies a rapidly developing country in which urbanization is replacing agricultural areas. Rapid development may allow the formation of foci where adequate sanitation is lacking, such as in markets. More aggressive intervention and vigilance by public health authorities to decrease the rat population in urban areas are warranted.

### Acknowledgments

We thank P. Slater and his staff from the Ministry of Health, Jerusalem, Israel, for their assistance in gathering the information.

Dr. Kariv is a specialist in internal medicine, completing a fellowship in gastroenterology and hepatology at Chaim Sheba Medical Center.

### References

1. Shenberg E, Gerichter B, Lindenbaum I. Leptospirosis in man: Israel 1970-1979. Am J Epidemiol 1982;115:352-8.
2. Lindenbaum I, Eylan E, Shenberg E. Leptospirosis in Israel: a report of 14 cases caused by Icterohemorrhagiae serogroup (1968-1982). Isr J Med Sci 1984;20:123-9.
3. Israel Center for Disease Control. Notifiable infectious diseases in Israel, 1951-1995. Tel Hashomer (Israel): The Center; 1996 Sept. Publication no. 201.
4. Lindenbaum I, Eylan E. Leptospirosis in *Rattus norvegicus* and *Rattus rattus* in Israel. Isr J Med Sci 1982;18:271-5.
5. Pate G, FitzSimon N, Mellotte GJ. Leptospirosis in the South-Eastern Health board region of the republic of Ireland: 1990 to 1996. Commun Dis Public Health 1999;2:217-8.
6. Centers for Disease Control and Prevention. Summary of notifiable diseases, United States, 1998. MMWR Morb Mortal Wkly Rep 1999;47:1-93.
7. Ciceroni L, Stepan E, Pinto A, Pizzocaro P, Dettori G, Franzin L, et al. Epidemiological trend of human leptospirosis in Italy between 1994-1996. Eur J Epidemiol 2000;16:79-86.
8. Olszyna DP, Jaspars R, Speelman P, van-Elzakker E, Korver H, Hartskeerl RA. Leptospirosis in the Netherlands. Ned Tijdschr Geneeskd 1998;142:1270-3.
9. Vinetz JM, Glass GE, Flexner CE, Mueller P, Kaslow DC. Sporadic urban leptospirosis. Ann Intern Med 1996;125:794-8.
10. Ko AI, Reis MG, Dourado CMR, Johnson WD, Riley LW, Salvador Leptospirosis study group. Urban epidemic of severe leptospirosis in Brazil. Lancet 1999;354:820-5.
